# Validity of the Favero Assioma Duo Power Pedal System for Measuring Power Output and Cadence

**DOI:** 10.3390/s21072277

**Published:** 2021-03-24

**Authors:** Almudena Montalvo-Pérez, Lidia B. Alejo, Pedro L. Valenzuela, Mario Castellanos, Jaime Gil-Cabrera, Eduardo Talavera, Alejandro Lucia, David Barranco-Gil

**Affiliations:** 1 Faculty of Sport Sciences, Universidad Europea de Madrid, 28640 Madrid, Spain; almudena.montalvo@universidadeuropea.es (A.M.-P.); pedroluis.valenzuela@universidadeuropea.es (P.L.V.); mcaste91@gmail.com (M.C.); jaime.gil@universidadeuropea.es (J.G.-C.); edutalfer@gmail.com (E.T.); alejandro.lucia@universidadeuropea.es (A.L.); david.barranco@universidadeuropea.es (D.B.-G.); 2 Instituto de Investigación Hospital 12 de Octubre (imas12), 28041 Madrid, Spain; 3 Department of Sport and Health, Spanish Agency for Health Protection in Sport (AEPSAD), 28016 Madrid, Spain

**Keywords:** cycling, pedal power meter, laboratory testing, power output, cadence

## Abstract

Cycling power meters enable monitoring external loads and performance changes. We aimed to determine the concurrent validity of the novel Favero Assioma Duo (FAD) pedal power meter compared with the crank-based SRM system (considered as gold standard). Thirty-three well-trained male cyclists were assessed at different power output (PO) levels (100–500 W and all-out 15-s sprints), pedaling cadences (75–100 rpm) and cycling positions (seating and standing) to compare the FAD device vs. SRM. No significant differences were found between devices for cadence nor for PO during all-out efforts (*p* > 0.05), although significant but small differences were found for efforts at lower PO values (*p* < 0.05 for 100–500 W, mean bias 3–8 W). A strong agreement was observed between both devices for mean cadence (ICC > 0.87) and PO values (ICC > 0.81) recorded in essentially all conditions and for peak cadence (ICC > 0.98) and peak PO (ICC > 0.99) during all-out efforts. The coefficient of variation for PO values was consistently lower than 3%. In conclusion, the FAD pedal-based power meter can be considered an overall valid system to record PO and cadence during cycling, although it might present a small bias compared with power meters placed on other locations such as SRM.

## 1. Introduction

A large number of cycling power meters have been developed over the last 20 years (e.g., SRM, Powertap Hub or Pedals, Stages) [1,2]. These devices allow cyclists and trainers to monitor work intensity in real time and to objectively assess their performance during training and competitions [3,4]. The cost of power meters has decreased considerably in recent years, thereby allowing most types of cyclists to use them.

The SRM crank (SRM, Jülich, Germany) is widely considered as the gold standard power meter (±1% accuracy) [5,6,7,8]. Other power meters such as the PowerTap hub (±1–3% reliability) [1,5,9], Stages crank (±1–3% reliability) [1,10,11], Garmin Vector pedals (±1–3% reliability) [1,12,13], and PowerTap P1 pedals (±1–2% reliability) [14,15,16] have been validated against SRM cranks. All these devices measure power output (PO) using strain gauges placed at distinct locations. For instance, SRM gauges are positioned between the crank and the chain rings [14], whereas the Stages crank is integrated into a small plastic case bonded to the rear side of the left crank arm [13], and the Powertap Hub has strain gauges located in the hub of the rear wheel [13]. On the other hand, most pedal-type power meters have the strain gauges located around the axle [13,14,17].

The rationale for locating the power meter in the pedals is that the PO of the athlete can be measured directly in the rider–bike interface, rather than in the point at which PO is transferred to the road (rear-hub-based system) [18], which does not consider the mechanical loss in the chain-drive system [1]. Instrumented pedals focus their technology on analyzing the applied torque, allowing the assessment of inter-relationships between the load and the effectiveness of the forces applied with both legs, and also the analysis of the thrust and braking forces in each pedal stroke [19]. These measurements permit the analysis of pedaling technique, which can be used to improve sports performance [20]. Moreover, the use of pedal power meters also provides flexibility in collecting PO data across a range of field conditions, as the pedals can be exchanged between different bikes (mountain bikes, road bikes, track bikes or time trial bikes).

The novel Favero Assioma Duo (FAD) pedal power meter bases its technology on using eight strain gauges placed around the axle to measure the slight deflections on the pedal spindle through the entire pedal stroke, as well as the two-dimensional force vectors [21]. In addition, the FAD uses a unique technology termed Instant Angular Velocity (IAV) Power System, which leverages an integrated gyroscope capable of detecting the IAV during the entire pedal stroke [21]. The FAD power meter also allows the examination of negative forces through the constant measurement of angular velocity, something that most devices do not measure, calculating the average angular velocity at every revolution [21]. Regarding practicality, the FAD power meter has some advantages over other devices that make it very attractive to cyclists, including its competitive price, its light weight, and the fact that it can be easily interchanged between different bikes compared to other devices.

Due to the widely spread use of pedal power meters, their potential advantage over other power meters, and particularly the growing popularity of the FAD power meter, the aim of the present study was to determine the concurrent validity of the FAD power meter under laboratory conditions when compared with SRM. We hypothesized that the FAD device would record similar PO values and cadence when compared with a scientifically validated system (SRM crank power meter [scientific model with 7075 extendable aluminum cranks: Schoberer Rad Messtechnik, Julich, Germany]).

## 2. Materials and Methods

### 2.1. Experimental Approach to the Problem

We conducted a descriptive cross-sectional study. Participants’ recruitment and data collection took place between October and December 2020. During a period of 4 weeks, each participant performed the tests in the same laboratory (exercise laboratory of the European University of Madrid, Spain) under standardized conditions (a temperature of 20.0 ± 1.1 °C and a humidity of 27.1 ± 1.6%). The study was conducted according to the tenets of the Declaration of Helsinki and was approved by the Investigation Ethics Commission of the European University of Madrid (CI-PI/20/165). Written informed consent was obtained from all participants prior to participation and after having the procedures explained in verbal and written form.

### 2.2. Participants

In total, 33 well-trained male cyclists participated in the study (age 28.8 ± 9.2 years; height 178.2 ± 5.3 cm; body mass 71.3 ± 8.6 kg; cycling training experience 5.2 ± 1.6 years). All participants had trained for 14.1 ± 3.0 h per week for a minimum of twelve months before the study. Participants were asked to avoid strenuous exercise, caffeine and alcohol for at least 24 h prior to testing sessions. Although an a priori sample size calculation was not performed, the sample size used was deemed appropriate based on the number of participants used in previous similar studies [14].

### 2.3. Procedures

**Figure 1:**
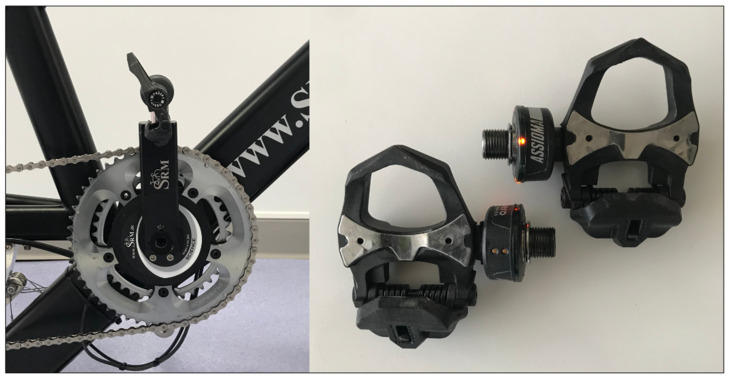
Representative example of the setting used on this study, placing the Favero Assioma Duo pedal power meter on an SRM crank.

A FAD power meter (Favero Electronics, srl., Arcade, TV, Italy) was compared against an SRM crank-based power meter (gold standard method), revised and calibrated following manufacturer’s instructions (scientific model with adjustable 7075 Aluminium crank length and 172.5-mm; Schoberer Rad Messtechnik, Julich, Germany) (Figure 1). The FAD power meter was mounted on the SRM cranks with the manufacturer’s recommended torque. An SRM indoor trainer was fitted with the SRM crank power meter. Data (cadence and PO) were recorded with a Garmin 520 cycling computer at a frequency of 1 Hz (Garmin International Inc., Olathe, KS, USA). Before the first trial, the pedals were fitted and set to the corresponding crank length of 172.5 mm in the Favero App. Furthermore, before each trial the recommended zero off-set calibration was performed on the FAD power meter according to the manufacturer’s recommendation. The bicycle saddle and handlebar position were individually adapted by the participants to ensure their comfort and mimic real-life conditions. In addition, cyclists used their own cycling shoes fitted with Look cleats.

**Figure 2:**
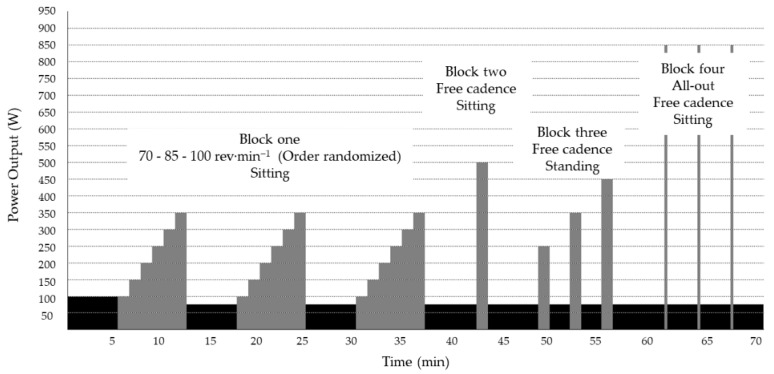
Graphical summary of the protocol.

The validity of the FAD was assessed in the laboratory using protocols derived from those used in previous studies [14,22]. We compared PO and cadence using different PO values, pedaling cadences and cycling positions (see Figure 2 for a graphical summary of the protocol). Participants performed a 5-min standardized warm-up at 100 W with a freely chosen cadence. The test consisted of 4 different blocks, interspersed by 5 min of recovery at 75 W performed in sitting position and with freely chosen cadence. Following the warm-up period, all participants began the first block, in which they performed three randomized and counterbalanced graded exercises periods, one for each of the selected fixed cadences (70, 85, and 100 rev·min^−1^) at six submaximal workloads (100, 150, 200, 250, 300, and 350 W) of 75 s duration. A metronome was used during this block to facilitate the cyclist’s adjustment to the required cadence [23]. After the first recovery period, cyclists performed block two, consisting of 75 s with a 500 W workload with free cadence and in a sitting position. They then performed another 5 min of recovery and started the third block, which consisted of three incremental loads (250, 350, and 450 W) during 75 s with a freely chosen cadence, in a standing pedaling position and with two minutes of recovery at 75 W, with freely chosen cadence between the three workloads. Finally, the cyclist executed block four, which consisted of three all-out sprints of 15 s in a sitting position interspersed by 3 min of recovery. Participants started the sprints with their dominant leg ready to pedal (crank at 0°).

PO and cadence data of the three initials blocks were collected for a minimum of 60 s. The initial 10 s and last 5 s from each data recording were discarded to allow sufficient time for the cyclists to stabilize and maintain each new load [6]. In the fourth block, consisting of three 15-s sprints, both the mean PO and cadence during the 15 s and the peak PO and peak cadence (highest values registered during one second) attained during the first 5 s were recorded [7,24].

### 2.4. Statistical Analysis

Data are presented as mean ± SD. The relationship and level of agreement between systems was analyzed with Pearson’s correlation coefficients (r), intra-class correlation coefficients (ICC), and typical errors of measurement (TEM), with all measures expressed together with 95% confidence interval (CI). r-values of 0.1, 0.3, 0.5, 0.7, and 0.9 were considered small, moderate, strong, very strong and extremely strong, respectively [25]. ICC values lower than 0.5, between 0.5 and 0.75, between 0.75 and 0.9, and greater than 0.90 were considered indicative of poor, moderate, good, and excellent agreement, respectively [26]. The coefficient of variation (CV) was calculated as the standard error of the mean expressed as a percentage of the mean, which provides an estimate of reliability [27]. Also, the TEM was expressed as a % of the mean of the measures, and used a measure of reliability with values lower than 10% considered reliable [28]. A two-way (system [FAD vs SRM] by PO) analysis of variance (ANOVA) for repeated measures was performed to determine differences between measurement systems at each specific cycling position (sitting/standing) and cadence. To reduce the risk of type I error, post hoc analyses (Bonferroni) were only performed when a significant system by PO interaction was found. The magnitude of the differences (effect size, ES) was analyzed using eta squared (ηp2). The limits of agreement (LoA) between systems are presented as bias ± 1.96 * SD. Statistical analyses were performed with a spreadsheet [29] and specific statistical software (SPSS 26.0, Inc., Chicago, IL, USA) setting the alpha for significance at 0.05.

## 3. Results

### 3.1. PO

#### 3.1.1. Validity

**Figure 3:**
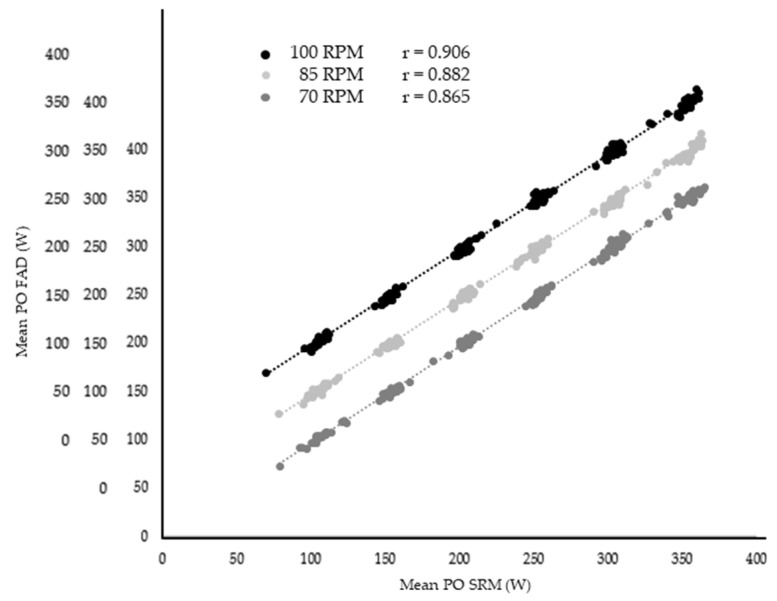
Pearson’s correlation coefficient between Favero Assioma Duo (FAD) and SRM power meter under three different cadences during block one. Abbreviation: PO, power output.

Significant but small differences in PO were found between FAD and SRM in BLOCK 1, 2 and 3 (*p* < 0.05 for PO by measurement system interactions, mean bias 3–8 W), with no significant differences were found during all-out efforts (*p* > 0.05) (Table 1). Strong-extremely strong r-values and ICCs were observed between the PO values recorded by the FAD and the SRM devices in all blocks (r > 0.81, ICC > 0.81) except for the seated position at 100 rev·min^−1^ and 300 W (r = 0.69, ICC = 0.66) (Figure 3, Table 1). Extremely strong r-values and ICCs were also found for the peak PO recorded during the sprints (r > 0.99, ICC > 0.99) (Table 2).

**Figure 4:**
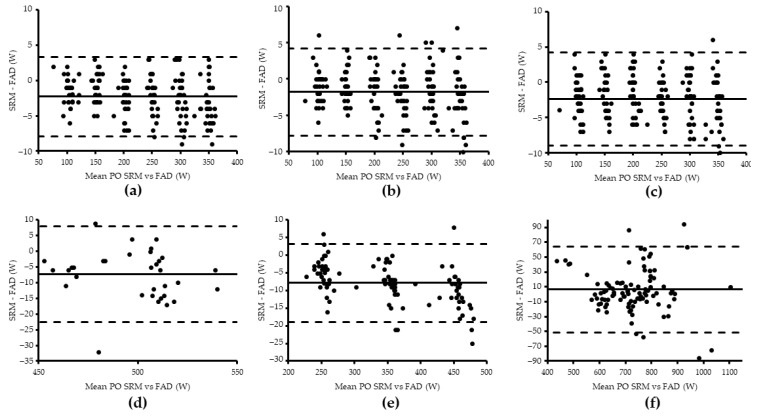
Bland–Altman plot representing the agreement between SRM and FAD mean power output (PO) for block one (**a**–**c**), two (**d**), three (**e**), and four (**f**).

**Figure 5:**
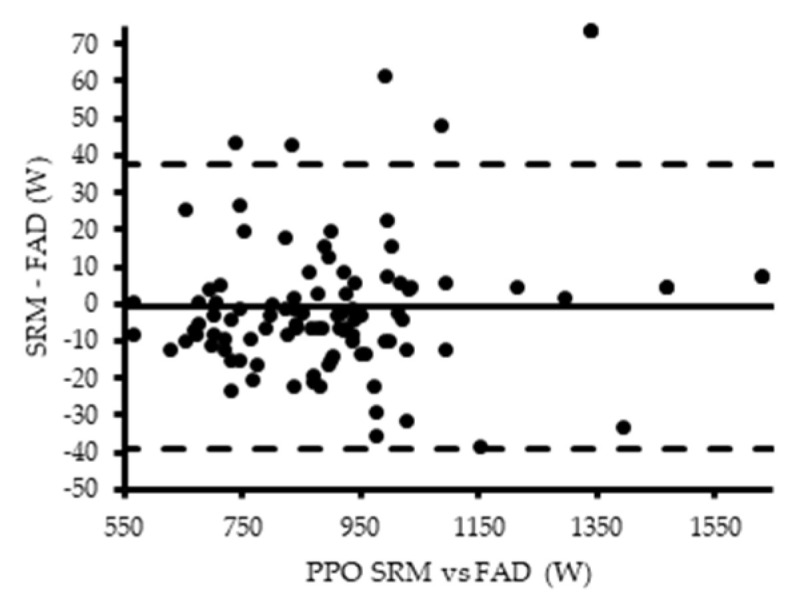
Bland-Altman plot representing the agreement between SRM and FAD peak power output (PPO) for block four.

There was a low bias between the PO values of the SRM and FAD in block one (−2.29 W at 70 rev·min^−1^, −1.78 W at 85 rev·min^−1^ and −2.41 W at 100 rev·min^−1^) (Figure 4a–c). A higher bias was detected in block two and three (−7.33 W and −7.86 W) (Figure 4d,e). The FAD pedals overestimated the mean PO data obtained by the SRM device except in block four (sprints) (6.26 W) (Figure 4f). There was a negligible bias between the peak PO values of the SRM and FAD in block four (−0.60 W) (Figure 5).

#### 3.1.2. Reliability

The CV% for mean PO values was lower than 1.84% in block one, two and three, 2.89% in block four, and 1.49% for peak PO during the sprints.

### 3.2. Cadence

#### 3.2.1. Validity

No significant measurement system by cadence interaction effect (*p* > 0.05) was found for any of the conditions (Table 3 and Table 4).

Strong-extremely strong r-values and ICCs were observed between the mean cadence values recorded by FAD and the SRM devices in all blocks (r > 0.87, ICC > 0.87). Extremely strong r-values and ICCs were found between the peak cadence in sprints (r > 0.98, ICC > 0.98).

#### 3.2.2. Reliability

The CV% for mean cadence was lower than 0.45% in block one, two and three, 1.10% in block four, and 0.83% for peak cadence during the sprints.

## 4. Discussion

The aim of the present study was to assess the validity of the FAD pedal power meter using a laboratory-based exercise protocol. The results of the study indicate that the FAD power meter system provides overall valid data for the measures of PO and cadence across a wide range of intensities. To our knowledge, this is the first study to assess the validity of this new power meter against the gold standard SRM device.

No significant differences were found between devices for cadence nor for PO during all-out efforts (*p* > 0.05), although significant but small differences were found for efforts at lower PO values (100–500 W). Particularly, we detected a small bias (i.e., <3 W for mean PO in block one (~1%) or <8 W in block three (~2%)) between the SRM and FAD for the mean PO data, with slightly higher values observed for FAD. However, this small bias is in line with the manufacturers’ instructions for calibration of other pedal-based power meters (Garmin Vector [1]), which state that the PO measured at the pedal can be higher than that measured by the SRM crank. This small bias might be due to the measurement location, and caused by PO losses in the bicycle components (e.g., mechanical material deformation of the crank, losses in the pedal–crank interface) [1]. Moreover, our results show a low CV% (<3% for all tests) and a high agreement (ICC > 0.80 for almost all measures) for the PO values registered with FAD and SRM, which support the validity of the FAD system for the measurement of PO. It must be noted that the typical error of measurements (in absolute units) increased in a directly proportional manner to the mean PO.

The importance of reproducible power meters to detect small changes in performance has been emphasized by Hopkins et al. [30]. Indeed, according to Hopkins [31], the CV in sports science reliability testing should not exceed 5%; in the present study, the FAD pedals CV% (compared with the SRM device) met this criterion for all tested PO values. The detectable change in performance represents a magnitude <2% in elite athletes [32] and the CVs obtained with the FAD in the present study are lower than this percentage with the exception of sprints (CV = 2.82%). Therefore, our findings allow users to be confident that their daily training results are consistently measured and that any observed changes in PO are real.

Our findings regarding the validity of the FAD power meter is in line with previous studies on other pedal power meters. Pallarés et al. [14] compared the Powertap P1 pedal power meter and SRM using the same protocol (without the sprint block) and found a similar bias (slightly higher values with the P1 pedals compared with SRM) and an extremely strong correlations (rho > 0.92). Similarly, a study by Czajkowski et al. [33] showed that the Powertap P1 pedals did non-significantly underestimate the PO during the submaximal incremental test (~1.5%). Moreover, the precision values reported for 54 power meters (pedal power metes, crank arms, crank spiders and wheel hub) in the study of Maier et al. [2] are in line with our results (CV < 2%). It is also important to note that in our study we found non-significant differences and a low bias for the mean (6.2 W, <0.7%) and peak PO values (0.6 W, 1.49%) recorded during the sprint block with FAD, being the latter a more reliable measure than the mean PO during sprints [34]. Moreover, we found a quasi-perfect correlation between the SRM and FAD devices for this outcome in the sprints block. The CV of the PO meters for block one (submaximal incremental test) shows similarities to previous studies comparing pedal power meters and the SRM device [1,22] and are consistent with the CV of the SRM obtained by Hutchinson et al. [35], whereas the CV of the Vector Pedals was higher. Likewise, in a study by Duc et al. [17], the CV of the ErgomoPro Power meter was higher than the CV of SRM and Powertap Hub.

The present study has some limitations that need to be mentioned, such as that all the tests were performed under laboratory conditions and with short measurements periods, which does not represent the changing ambient conditions and longer recording periods that usually occur in training sessions and competitions. Also, subjects were not familiarized with the protocol before the test. Future studies should compare the PO between the FAD and the SRM crank set during real cycling on the field to assess the sensitivity of the power meter considering road vibrations. Moreover, the protocol we used was not specifically designed to assess the test-retest reliability of the systems. Given that PO was not fixed, the reliability of the PO measures during each condition depended on both the reliability of the systems (SRM and FAD) and the inner biological variability of the participants while pedaling. This resulted in a lower reliability when pedaling at very low intensities (e.g., CV > 10% for both SRM and FAD when trying to pedal at 100 W) but higher reliability at higher intensities (e.g., CV < 3% when pedaling at 150–300 W). However, similar CVs were found between FAD and the gold standard SRM, and the overall CV (the comparison between FAD and SRM) values were low (<3%). Finally, we found an overall strong correlation between FAD and SRM for all PO values except for 300 W at 100 rpm (r = 0.69). This might have been caused by the small variations among subjects when pedaling at ~300 W (as supported by the low CV values [<1.8%] found for both SRM and FAD]), which hinders observing strong correlations at that specific PO, and indeed when attending at the whole range of PO values the correlation between both devices was strong (r > 0.87).

In summary, the present findings overall support the concurrent validity of the FAD pedal power meter compared with SRM, although the former can result in slightly higher PO values (3–8 W on average) that might be potentially due to the measurement location (pedal vs. crank). Therefore, the PO values registered between these devices should not be used interchangeably. The novel FAD power meter might offer some advantages over other power meters as it measures power in the direct zone of application (the pedal), is cheaper than other power meters, can be easily interchanged between bikes and can be used to assess the PO of each leg separately.

## 5. Conclusions

The FAD pedal power meter can be considered an overall valid device to record PO and cadence in different conditions, presenting an overall good agreement (ICC > ~0.80) and low albeit significant bias (<~8 W and <~2% for all tests) compared to the PO values registered by the gold standard SRM device.

## Figures and Tables

**Table 1:** Analysis of the mean power output (PO) registered by the SRM and the FAD power meters.

			SRM (W)		FAD (W)		TEM (W)	CV%		ICC	Pearson r-Value	PO × Measurement System *p*-Value	Effect Size (η_p_^2^)	Bland-Altman	
			Mean ± SD	CV	Mean ± SD	CV								Bias (W)	SD Bias (W)
70 rev·min^−1^	Sitting	100 W	104.5 ± 11.3	10.84%	106.0 ± 11.6 ***	10.91%	1.34	1.27%	0.89%	0.99	0.99	0.006	0.121	−2.29	2.86 LoA (−7.89 to 3.31)
		150 W	151.5 ± 4.1	2.71%	152.6 ± 3.7 **	2.44%	1.35	0.89%		0.89	0.90				
		200 W	200.2 ± 5.5	2.73%	202.8 ± 5.5 ***	2.71%	1.65	0.82%		0.91	0.93				
		250 W	249.1 ± 3.6	1.45%	251.1 ± 5.2 ***	2.06%	1.90	0.76%		0.83	0.88				
		300 W	298.9 ± 5.4	1.81%	301.9 ± 7.3 ***	2.41%	2.68	0.89%		0.83	0.84				
		350 W	346.9 ± 7.6	2.19%	350.5 ± 7.8 ***	2.24%	2.38	0.68%		0.91	0.89				
85 rev·min^−1^	Sitting	100 W	102.2 ± 6.8	6.67%	103.2 ± 6.8 *	6.61%	1.63	1.59%	1.01%	0.95	0.94	0.020	0.091	−1.78	3.05 LoA (−7.76 to 4.20)
		150 W	150.4 ± 3.5	2.31%	151.5 ± 3.6 **	2.39%	1.51	1.00%		0.83	0.81				
		200 W	199.8 ± 4.4	2.19%	201.8 ± 5.8 ***	2.89%	2.11	1.05%		0.84	0.88				
		250 W	247.3 ± 5.6	2.26%	249.4 ± 6.7 ***	2.69%	2.11	0.85%		0.89	0.90				
		300 W	298.0 ± 4.4	1.47%	299.6 ± 5.6 **	1.88%	2.08	0.70%		0.84	0.85				
		350 W	347.1 ± 8.2	2.35%	345.0 ± 10.3 ***	2.94%	3.02	0.87%		0.90	0.92				
100 rev·min^−1^	Sitting	100 W	101.9 ± 7.1	6.98%	104.0 ± 7.8 ***	7.50%	1.89	1.84%	1.15%	0.94	0.92	0.020	0.088	−2.41	3.37 LoA (−9.02 to 4.20)
		150 W	149.9 ± 3.7	2.47%	151.8 ± 5.2 ***	3.46%	1.99	1.32%		0.82	0.86				
		200 W	99.8 ± 3.9	1.95%	201.3 ± 5.2 **	2.56%	2.07	1.03%		0.81	0.87				
		250 W	248.6 ± 6.1	2.44%	251.6 ± 6.4 ***	2.53%	2.39	0.96%		0.86	0.86				
		300 W	298.8 ± 3.8	1.28%	301.5 ± 5.4 ***	1.78%	2.78	0.93%		0.66	0.69				
		350 W	338.5 ± 44.7	13.19%	341.9 ± 44.5 ***	13.03%	2.88	0.85%		0.99	0.99				
FCC	Sitting	500 W	494.0 ± 22.5	4.56%	501.4 ± 23.6 ***	4.72%	5.51	1.11%	1.11%	0.95	0.94	<0.001	0.481	−7.33	7.80 LoA (−22.62 to 3.27)
FCC	Standing	250 W	250.2 ± 7.8	3.11%	255.1 ± 8.9 ***	3.48%	3.18	1.26%	1.06%	0.86	0.86	<0.001	0.803	−7.86	5.68 LoA (−18.99 to 3.27)
		350 W	345.8 ± 17.1	4.95%	353.4 ± 18.2 ***	5.27%	3.42	0.98%		0.97	0.97				
		450 W	442.2 ± 27.8	6.30%	453.2 ± 28.0 ***	6.31%	4.23	0.94%		0.98	0.97				
FCC	Sitting	ALL-OUT	756.3 ± 143.0	18.90%	755.0 ± 155.4	20.60%	21.87	2.89%	2.82%	0.98	0.94	0.167	0.057	6.26	29.39 LoA (−51.34 to 63.86)
		ALL-OUT	736.3 ± 114.5	15.56%	723.1 ± 100.3	13.96%	21.03	2.88%		0.96	0.97				
		ALL-OUT	715.1 ± 102.0	14.26%	710.7 ± 103.3	14.53%	19.03	2.67%		0.97	0.97				

Abbreviations: CV, coefficient of variation; CV%, percentage of the TEM; FCC, freely chosen cadence; LoA, Limits of Agreement; SD, standard deviation; TEM, typical error of measurement. Significant differences compared to SRM: * *p* < 0.05, ** *p* < 0.01, *** *p* < 0.001.

**Table 2:** Analysis of the peak power output (PPO) registered by the SRM and the FAD power meters during all-out sprints.

			SRM (W)		FAD (W)		TEM (W)	CV%		ICC	Pearson r-Value	PO × Measurement System *p*-Value	Effect Size (η_p_^2^)	Bland-Altman Analysis	
			Mean ± SD	CV	Mean ± SD	CV								Bias (W)	SD Bias (W)
FCC	Sitting	ALL-OUT	947.2 ± 233.6	24.67%	943.6 ± 233.1	24.71%	14.65	1.55%	1.49%	0.99	0.99	0.282	0.039	−0.60	19.63 LoA (−39.08 to 37.89)
		ALL-OUT	898.4 ± 204.4	22.75%	900.9 ± 203.9	22.64%	9.10	1.01%		0.99	0.99				
		ALL-OUT	877.0 ± 189.0	21.56%	879.8 ± 178.4	20.28%	16.71	1.90%		0.99	0.99				

Abbreviations: CV, coefficient of variation; CV%, percentage of the TEM; FCC, freely chosen cadence; LoA, Limits of Agreement; PPO, peak power output; SD, standard deviation; TEM, typical error of measurement.

**Table 3:** Analysis of the mean cadence registered by the SRM and the FAD power meters.

			SRM (rev·min^−1^)		FAD (rev·min^−1^)		TEM (rev·min^−1^)	CV%		ICC	Pearson r-Value	Cadence × Measurement System *p*-Value	Effect Size (ηp^2^)
			Mean ± SD	CV	Mean ± SD	CV							
70 rev·min^−1^	Sitting	100 W	70.7 ± 2.0	2.77%	70.9 ± 1.9	2.73%	0.31	0.44%	0.42%	0.88	0.98	0.164	0.048
		150 W	70.0 ± 1.4	2.05%	70.1 ± 1.4	1.96%	0.26	0.37%		0.97	0.97		
		200 W	70.4 ± 1.4	2.04%	70.8 ± 1.3	1.90%	0.35	0.50%		0.94	0.94		
		250 W	70.2 ± 1.4	2.01%	70.5 ± 1.4	2.04%	0.32	0.45%		0.95	0.95		
		300 W	70.2 ± 1.4	2.05%	70.4 ± 1.4	2.01%	0.26	0.37%		0.97	0.97		
		350 W	70.6 ± 1.6	2.29%	70.8 ± 1.7	2.41%	0.26	0.37%		0.98	0.98		
85 rev·min^−1^	Sitting	100 W	84.0 ± 1.8	2.11%	84.4 ± 1.9	2.20%	0.35	0.42%	0.38%	0.96	0.96	0.190	0.046
		150 W	84.4 ± 1.0	1.15%	84.7 ± 1.0	1.13%	0.31	0.37%		0.90	0.90		
		200 W	84.4 ± 0.9	1.10%	84.6 ± 0.9	1.10%	0.32	0.38%		0.89	0.88		
		250 W	84.4 ± 0.9	1.11%	84.7 ± 0.9	1.07%	0.34	0.40%		0.87	0.87		
		300 W	84.5 ± 1.5	1.78%	84.8 ± 1.5	1.81%	0.35	0.41%		0.95	0.95		
		350 W	85.0 ± 2.0	2.33%	85.1 ± 2.0	2.41%	0.26	0.31%		0.98	0.98		
100 rev·min^−1^	Sitting	100 W	98.8 ± 1.9	1.94%	99.2 ± 2.0	2.02%	0.35	0.35%	0.36%	0.97	0.97	0.900	0.009
		150 W	99.0 ± 1.9	1.97%	99.3 ± 1.8	1.86%	0.34	0.34%		0.97	0.97		
		200 W	98.9 ± 2.5	2.49%	99.3 ± 2.3	2.36%	0.35	0.35%		0.98	0.98		
		250 W	99.3 ± 1.8	1.86%	99.7 ± 1.8	1.83%	0.37	0.37%		0.96	0.96		
		300 W	99.2 ± 2.0	1.98%	99.7 ± 2.1	2.09%	0.36	0.36%		0.97	0.97		
		350 W	99.7 ± 3.5	3.50%	100.1 ± 3.4	3.45%	0.36	0.36%		0.99	0.99		
FCC	Sitting	500 W	91.3 ± 7.3	8.03%	91.7 ± 7.2	7.91%	0.35	0.38%	0.38%	0.99	0.99		
FCC	Standing	250 W	77.1 ± 8.2	10.69%	77.4 ± 8.3	10.76%	0.33	0.43%	0.45%	0.99	0.99	0.580	0.017
		350 W	77.9 ± 6.4	8.26%	78.3 ± 6.6	8.44%	0.39	0.50%		0.99	0.99		
		450 W	82.2 ± 4.6	5.58%	82.6 ± 4.5	5.48%	0.35	0.42%		0.99	0.99		
FCC	Sitting	ALL-OUT	115.8 ± 11.4	9.88%	115.0 ± 11.4	9.95%	1.43	1.24%	1.10%	0.99	0.98	0.512	0.020
		ALL-OUT	116.7 ± 9.8	8.44%	116.0 ± 9.5	8.20%	1.20	1.03%		0.99	0.99		
		ALL-OUT	115.8 ± 9.8	8.49%	115.4 ± 9.7	8.44%	1.20	1.04%		0.99	0.99		

Abbreviations: CV, coefficient of variation; CV%, percentage of the TEM; FCC, freely chosen cadence; LoA, Limits of Agreement; SD, standard deviation; TEM, typical error of measurement.

**Table 4:** Analysis of the peak cadence registered by the SRM and the FAD power meters during all-out sprints.

			PEAK CADENCE										
			SRM (rev·min^−1^)		FAD (rev·min^−1^)		TEM (rev·min^−1^)	CV%		ICC	Pearson r-Value	Cadence × Measurement System *p*-Value	Effect Size (η_p_^2^)
			Mean ± SD	CV	Mean ± SD	CV							
FCC	Sitting	ALL-OUT	129.8 ± 17.9	13.79%	129.9 ± 17.7	13.61%	0.79	0.61%	0.83%	0.99	0.99	0.299	0.036
		ALL-OUT	130.5 ± 13.8	10.55%	130.8 ± 13.8	10.54%	0.84	0.64%		0.99	0.99		
		ALL-OUT	128.4 ± 12.2	9.51%	129.1 ± 11.8	9.12%	1.61	1.25%		0.98	0.98		

Abbreviations: CV, coefficient of variation; CV%, percentage of the TEM; FCC, freely chosen cadence; LoA, Limits of Agreement; SD, standard deviation; TEM, typical error of measurement.
